# Rates of delirium referrals to the Neuropsychiatry Service in a tertiary referral centre hospital

**DOI:** 10.1192/j.eurpsy.2024.236

**Published:** 2024-08-27

**Authors:** S. M. Gunawardena, S. Riaz, K. Murphy

**Affiliations:** Neuropsychiatry, Beaumont Hospital, Dublin, Dublin, Ireland

## Abstract

**Introduction:**

Beaumont Hospital is the National Neurosurgical Centre in Ireland. Due to the high numbers of referrals from Neurology and Neurosurgery, The Department of Psychiatry established a specialist Neuropsychiatry inpatient Liaison service and a weekly Neuropsychiatry outpatient clinic. Many of the referrals that the service receive involve the management of delirium. Delirium is a common medical complication, particularly in neurosurgical settings. Delirium causes significant symptom burden which can lead to distress to all involved and impacts quality of life.

**Objectives:**

The aim was to improve the rates of referrals for delirium presentations and referral rates overall from Neurology and Neurosurgery. The neuropsychiatry service have implemented a delirium protocol for all medical and surgical teams in Beaumont Hospital. This protocol can be accessed through the Beaumont hospital phone app, or on site on each ward. For this reason, delirium can be managed by medical teams in the first instance. If this is not successful, neuropsychiatry can be contacted for further advice or review of patients with more complicated presentations.

**Methods:**

The neuropsychiatry service receives referrals through the Patient Information Profile Explorer system which is accessed through the Beaumont Hospital online portal. In the event of an urgent referral, neurology or neurosurgery teams can contact the neuropsychiatry service directly by phone. Referrals are logged on the team referral log book, and details of the referral are recorded along with diagnosis and management. Data was collected retrospectively from the PIPE and log book to measure the rates and reasons for referrals over a one year period. Rates and details of referrals were initially recorded between July-December 2022. An educational intervention was provided where psychoeducation was provided to junior hospital doctors during protected teaching times and further education was provided over the phone when referrals were discussed between team members. Rates and details of referrals were then recorded between January-July 2023.

**Results:**

There was a reduction in referrals when comparing the two six month periods. There were 115 neuropsychiatry referrals from July to December 2022 and 78 referrals from January to July 2023. Rates of delirium referrals also reduced from 31% to 25% after psychoeducation was provided to junior doctors.

**Conclusions:**

This audit highlights the importance of communication and education for medical and surgical trainees in the management of delirium. There is a high rate of turnover of junior doctors throughout the year in Beaumont Hospital. For this reason, it is imperative that continued education is provided to allow them to follow the delirium protocol independently before seeking tertiary service assistance. Ultimately, early and rapid intervention of delirium can have a positive impact on patient care and prognosis

**Disclosure of Interest:**

None Declared

